# Personality traits and risk of eating disorders among Polish women: the moderating role of self-esteem

**DOI:** 10.3389/fpsyt.2023.1281898

**Published:** 2023-10-31

**Authors:** Kamila Rachubińska, Anna Maria Cybulska, Daria Schneider-Matyka, Mariusz Panczyk, Ewa Kupcewicz, Magdalena Kamińska, Elżbieta Grochans

**Affiliations:** ^1^Department of Nursing, Faculty of Health Sciences, Pomeranian Medical University in Szczecin, Szczecin, Poland; ^2^Department of Education and Research in Health Sciences, Faculty of Health Science, Medical University of Warsaw, Warsaw, Poland; ^3^Department of Nursing, Collegium Medicum, University of Warmia and Mazury in Olsztyn, Olsztyn, Poland; ^4^Subdepartment of Long-Term Care and Palliative Medicine, Department of Social Medicine, Faculty of Health Sciences, Pomeranian Medical University in Szczecin, Szczecin, Poland

**Keywords:** behaviors, eating disorders, orthorexia nervosa, personality traits, self-esteem

## Abstract

**Objectives:**

Personality traits should be taken into account when diagnosing individuals with disordered eating behaviors in the hope of better understanding their etiology and symptom progression and when planning treatment. The objective of this study was to attempt to determine the moderating role of self-esteem in the relationships between personality traits included in the Big Five model among Polish women and estimated risk of eating disorders.

**Methods:**

The study was conducted among 556 Polish women from Zachodniopomorskie Voivodeship. The average age of the women under study was 34 years. A diagnostic survey was used as the research method, and the empirical data were collected using the following research tools: The NEO Five-Factor Inventory (NEO-FFI), Rosenberg Self-Esteem Scale (SES), ORTO – 15 *Questionnaire*, The Three-Factor Eating Questionnaire (TFEQ-13), and the authors’ original questionnaire. A multivariate linear regression analysis was conducted to examine the influence of selected independent variables. The assumptions for the linear regression model were satisfied, as indicated by the Ramsey Regression Equation Specification Error Test, White’s test, and the Jarque-Bera test.

**Results:**

Only the personality trait of neuroticism exhibits a statistically significant effect on the “Cognitive Restraint of Eating,” “Uncontrolled Eating,” and “Emotional Eating” scores (*p* < 0.001). The moderation effect was demonstrated between self-esteem and the personality trait of conscientiousness on the “Cognitive Restraint of Eating” scale score. There is a moderation effect between self-esteem and the personality trait of extraversion on the “Uncontrolled Eating” subscale score. There is a moderation effect between self-esteem and the personality trait of conscientiousness on the “Uncontrolled Eating” scale score.

**Conclusion:**

Self-esteem was not a predictor of the occurrence of risk of eating disorders while playing a moderating role in the relationship between certain personality traits and estimated risk of eating disorders. A higher level of neuroticism was identified as an important predictor of higher results for orthorexia, Cognitive Restraint of Eating, Uncontrolled Eating, and Emotional Eating. It was also demonstrated that the orthorexia risk index decreased with increased extraversion and openness to experience. The results of this study suggest that eating behaviors and psychological factors should be included in psychological interventions in the treatment of eating disorders. The clinical goal can be considered to be an improvement in non-normative eating behaviors, such as a reduction in overeating episodes or eating less frequently in the absence of a hunger feeling. In order to assist these individuals in their attempts to achieve healthy behaviors, variables related to mental functioning can be then identified as important goals to support individuals in their efforts to change health behaviors by achieving better mental well-being.

## 1. Introduction

Eating behavior is a complex construct determined by a number of factors: from sex, age, body weight, climatic, sociocultural, and religious factors to individual lifestyle preferences, psychological factors and habits (1, 2). From an early age, the individual acquires specific food consumption behaviors (3). Eating food begins to play many roles in people’s lives, including those related to the psychological sphere (4). Disordered eating refers to troublesome eating behaviors such as the use of restrictive diets, overeating, emotional eating, or the use of laxatives. These disorders are less severe than those required to meet the full DSM V criteria. They can be considered as a result of complex problems such as random, unfavorable life events, family situations, failures at work, or abnormalities in feeling and expressing emotions. Eating disorders can be determined by numerous family, cultural, and individual factors (3). Eating food can replace the need for love or help relieve boredom. Anger, sadness, fear, happiness or loneliness can change the existing eating behaviors and direct a particular individual’s eating choices differently (5). A lack of ability to control one’s own emotions and stress can adversely affect the individual. Attempting to reduce tension by eating often results in overweight and even obesity (6). Emotional eating is associated with increased food consumption under the influence of emotions (7). Under the influence of strong emotions, both positive and negative, an individual’s control over the amount of food they eat can be reduced. The lack of restrictions on the amount of food consumed results in the disturbance of proper eating habits, which may adversely affect the health of a particular individual (8). In population studies, one can only track the occurrence of the initial symptoms of abnormal eating behaviors which may or may not lead to the clinical manifestation of serious health problems such as overweight or obesity, anorexia, or bulimia (7, 8). As emphasized in the title, the current study is focused on disordered eating regarded as a behavior and not a disease.

Orthorexia nervosa is also considered a part of eating behaviors. The central point in the occurrence of orthorexia is the attempt to achieve optimal health through strict dietary control (9). Individuals affected by orthorexia focus on the quality of the products they consume instead of the quantity and spend a lot of time checking the sources of food, meal preparation methods, food processing, the content of preservatives, and materials used for food packaging. Other important aspects of orthorexia include the need to collect foodstuffs, paying attention to weighing and measuring them, planning meals in advance, and the occurrence of intrusive thoughts during the time not devoted to nutrition-related activities (10). The daily routine of patients, divided into four phases, has been described. The first of them is excessive thinking about what food will be eaten today or the next day. The next phase involves collecting individual food ingredients with a very critical approach and considerable control. The third phase involves the preparation of meals in the healthiest way possible. The last phase is a feeling of satisfaction or guilt, depending on the course of the preceding phases (11).

To date, orthorexia nervosa (ON) has not been included in any of the official diagnostic classifications (12–14). It is debated whether ON is a separate set of symptoms or is linked to existing clinical syndromes. Some authors assume that ON symptoms are not a consequence of other mental disorders (15–17) or the process of treating somatic conditions (18). At the same time, there are suggestions that ON is associated with features of obsessive-compulsive disorder (OCD) (19), eating disorders (ED) (20), and anxiety disorders (21). Most studies indicate that sex does not increase the severity of orthorexia features (22). Some authors, however, report a higher prevalence of orthorexia symptoms among women (23).

An assessment of eating behaviors and the related factors, based on self-report tools, can be distorted due to the desire to be perceived by society as attractive, i.e., a tendency to avoid criticism and provide more socially acceptable answers (24). This may result in overestimating healthy behaviors and underestimating undesirable ones (25, 26). Knowledge of the effect of social desirability on self-reported eating behaviors and their links with other factors may be useful in improving the accuracy of dietary assessment and developing effective strategies for preventing eating and body weight disorders.

Previous studies conducted in European countries demonstrated that the levels of eating behavior dimensions varied depending on the sex (27). In general, research demonstrated that women score higher in terms of cognitive restraint uncontrolled eating than men. Recent studies also confirm that sex, age and educational background are linked to cognitive restraint uncontrolled eating and emotional eating (28).

Many women consciously try to limit their food intake to achieve or maintain their desired body weight. This is called Cognitive Restraint of Eating and is a type of eating behavior that is regulated by cognitive processes and not physiological mechanisms such as hunger and satiety (29). Although there are several scales for the assessment of Cognitive Restraint of Eating, the Three-Factor Eating Questionnaire (TFEQ) scale (30) is regarded as being robust and exhibits good psychometric properties (31). Usually, women with high Cognitive Restraint of Eating scores are very conscious of the amount and type of the foods they consume, although reports vary as to whether their energy intake is actually lower than that in women with low Restraint of Eating scores (32).

Women usually tend to be more concerned about body weight and may be more prone to restrict their food intake to control their weight. They are also more likely to eat in response to stress and negative emotions. The results suggest that men appear to be more likely to eat large amounts of food, which may be due to the palatability of food, hunger, and social signals (2). Similar sex differences in the TFEQ subscale results were identified among French adolescents and adults, with the exception of Uncontrolled Eating in middle-aged adults (33). Higher scores for the Emotional and Cognitive Restraint of Eating among female students were also noted in Portugal (27). Studies conducted among Lebanese and Chilean students also demonstrated that women scored higher on Emotional Eating (34). In contrast, a study conducted among adult Germans demonstrated that women scored higher on all three dimensions of eating behaviors (TFEQ subscales) (35). de Castro (36) observed sex differences in factors motivating cognitive dietary restrictions. In women, cognitive restraint appeared to be related to the fear of gaining weight, while the actual attempt to lose weight would motivate restrictive behavior in men (28).

Some researchers argued that personality traits should be taken into account when diagnosing individuals with disordered eating behaviors in the hope of better understanding their etiology and symptom progression and when planning treatment (37). According to Bollen and Wojciechowski (38), very few studies used the Five-Factor Model in this field of research. However, it was suggested that this model could provide a lot of relevant information regarding the diagnosis of eating disorders (39). In addition, as previously asserted by Elfhag and Morey (40), further research is needed on larger samples of women who are preoccupied with their body weight. This is because individuals who seek help from professionals appear to be more emotionally depressed and dissatisfied with their body image than others (15). Therefore, mental and behavioral problems in the female population should be studied more extensively.

Eating disorders are an important mental health issue due to their overall prevalence. Low self-esteem is considered, like others, to be one of the risk factors for the development of these disorders, with no explanation of the actual impact of low self-esteem on the development of eating disorders (16). Measurement of personality traits and sel-esteem can be useful to identify women who can be prone to non-normative eating behaviors, which, in turn, may hinder their attempts to control their body weight. Eating behaviors and psychological factors should be included in psychological interventions in the treatment of eating disorders. The clinical goal can be considered to be an improvement in non-normative eating behaviors, such as a reduction in overeating episodes or eating less frequently in the absence of a hunger feeling. In order to assist these individuals in their attempts to achieve healthy behaviors, variables related to mental functioning can be then identified as important goals to support individuals in their efforts to change health behaviors by achieving better mental well-being. The main objective of the study was to attempt to determine the moderative role of self-esteem in the relationships between personality traits included in the five-factor model known as the Big Five model among Polish women and estimated risk of eating disorders.

## 2. Materials and methods

### 2.1. Research design

The study was conducted among 556 women from Zachodniopomorskie Voivodeship (Poland). When selecting the sample for the study, a sample size calculator from the statistical software program STATISTICA version for Windows 13.1 by TIBCO Software Inc. – StatSoft, Poland, was used with a 95% confidence interval. This ensured that the sample is representative. Based on the data regarding the number of women residing in the West Pomeranian Voivodeship, the minimum patient group that should be included in the study is 384 individuals. The selection of the group was random. No randomizing tool was used. It was based on self-reported participants who met the inclusion criteria as described in the limitations. The criteria for inclusion in the study were as follows: the age >18 years, female sex, place of residence in Zachodniopomorskie Voivodeship, no diagnosed mental disorders, granting informed, written consent to participate in the study, and the completion of the provided set of questionnaires. The criteria for exclusion were as follows: the age <18 years, place of residence other than West Pomeranien Voivodeship, diagnosed mental disorders, failure to provide written consent and not filling out the form completely. A total of 605 women were invited to participate in the survey. Only 556 women correctly completed the surveys (completion rate: 92%). Before the commencement of the project, the approval of the Research Ethics Committee of the Pomeranian Medical University in Szczecin was obtained (Resolution No KB-0012/518/12/16). After obtaining permission to conduct the study, pre-prepared research tool sheets were distributed personally by the author of the article to the women who had read the above-mentioned information and agreed to participate in the project. The female participants were informed of the objective of the study and anonymity and had the opportunity to ask questions and receive comprehensive explanations. The participants could opt out of the study at any time without giving a reason. The research and trained interviewers were carried out using the traditional method, by disseminating paper versions of questionnaires among women from the West Pomeranian Voivodeship. The study is part of a larger research project.

### 2.2. Surveys

The study was conducted by the diagnostic survey method using a questionnaire technique. In order to analyse the role of self-esteem in relationships between the personality traits included in the five-factor model and among Polish women and estimated risk of eating disorders, both the following standardized tools adapted to the Polish conditions and the original authors’ questionnaire were used.

#### 2.2.1. The NEO five-factor inventory

The patients’ personality traits were assessed using the NEO five-factor inventory (NEO-FFI) questionnaire developed by Costa and McCrae (17). The questionnaire was adapted to the Polish conditions by Zawadzki et al. (41). This method includes 60 statements. The participant is asked to refer to each of them by indicating on a 5-point scale (from 1 - “I strongly disagree” to 5 - “I strongly agree”) the answer that is most in line with their beliefs. The raw scores are converted into sten scores and expressed by five factors according to the so-called Big Five model, which measures neuroticism (N) (Cronbach’s alpha 0.890), extraversion (E) (Cronbach’s alpha 0.807), openness to experience (O) (Cronbach’s alpha 0.798), agreeableness (A) (Cronbach’s alpha 0.763), and conscientiousness (C) (Cronbach’s alpha 0.855). The obtained value of Cronbach’s α statistics is 0.924.

#### 2.2.2. Rosenberg self-esteem scale

It is a tool for assessing the level of general self-esteem, understood as a conscious attitude toward one’s own self and a belief in one’s own worth, revealed in a self-report and regarded as a relatively constant characteristic of a particular individual. The scale comprises 10 diagnostic statements, and the participant indicates, using a four-point scale, the extent to which they agree with each statement. The Polish self-esteem scale (SES) adaptation is adequate, as confirmed by the results obtained, e.g., by the application of the exploratory and confirmatory factor analysis and the correlation of SES with questionnaires that measure other constructs (such as temperament, locus of control, social competence, etc.). The scale was adapted to the Polish conditions by Łaguna et al. (42) and (43). Alpha-Cronbach’s internal consistency coefficient was 0.888.

#### 2.2.3. ORTO-15 questionnaire

The ORTO-15 questionnaire developed by Donini et al. (19) is the most commonly used research tool to assess the intensity of the orthorexia nervosa (ON) symptoms - the Orthorexia Risk Index (ORI). Its questions refer to the cognitive, emotional, and clinical aspects of ON. The validation of ORTO-15 under Polish conditions was conducted by a team headed by Stochel et al. (44). The obtained value of Cronbach’s α statistics is 0.627.

#### 2.2.4. The three-factor eating questionnaire

The questions provided in the TFEQ-13 questionnaire are indexed in three subscales that measure the cognitive-behavioral and emotional aspects of eating behaviors. The first subscale measures behaviors related to restricting the quantity or type of food in order to control body weight and body image. The second one measures the tendency to eat more than usual due to loss of control over eating or an incontrollable feeling of hunger that triggers overeating binges. The third subscale measures overeating episodes caused by feelings of depressed mood and anxiety. The questions of the TFEQ-13 scale concern three factors: (1) Cognitive Restraint of Eating, (2) Uncontrolled Eating, and (3) Emotional Eating. The validation of ORTO-15 under Polish conditions was conducted by Stunkard and Messick (30) and Dzielska et al. (45). Alpha-Cronbach’s internal consistency coefficient was 0.821.

#### 2.2.5. The original authors’ questionnaire

The authors’ survey questionnaire included closed and semi-open-ended questions aimed at acquiring selected sociodemographic data for the participants, i.e., age, educational level, marital status, place of residence, and professional activity.

### 2.3. Statistical analysis

Descriptive statistics, including the mean (M), standard deviation (SD), median (Me), quartile range (Q1–Q3), and range (Min–Max), were computed for each variable. The skewness of the distribution was also assessed by calculating the asymmetry coefficient, as a regression model was employed in subsequent stages of the analysis.

A multivariate linear regression analysis was conducted to examine the influence of selected independent variables, namely the severity of five distinct personality traits and self-esteem, on the orthorexia risk index and the cognitive-behavioral and emotional aspects of eating behaviors. The assumptions for the linear regression model were satisfied, as indicated by the Ramsey Regression Equation Specification Error Test, White’s test, and the Jarque-Bera test. The presence of autocorrelation was also evaluated by calculating the variance inflation factor (VIF).

The moderation effect was analyzed, with self-esteem serving as a moderator in the relationship between the selected personality trait and the cognitive-behavioral and emotional aspects of eating behaviors. A moderator describes the level of change between the independent and dependent variables, and it is quantified by the linear regression coefficient of the product term.

The parameters of the regression models were estimated using the least squares method, and a standardized regression coefficient with a 95% confidence interval was determined for each independent variable.

Statistical significance was set at a *p*-value less than 0.05. All analyses were performed using STATISTICA 13.3 software (TIBCO Software, Palo Alto, California, United States).

## 3. Results

The study involved 556 female participants from Zachodniopomorskie Voivodeship. The average age of the women under study was 34 years. Slightly more than half of the participants had lower education (51.6%) and resided in a locality with a population of less than 100,000 inhabitants (52.3%). Most women reported being in a relationship (66.5%) and being professionally active (89.2%) (Table S1). The orthorexia risk index was assessed using the ORTO-15. The mean score was 37.45 ± 5.395. On the other hand, the TFEQ-13 questionnaire assessed the following factors: Cognitive Restraint of Eating - the mean score was 6.2 ± 2.9, Uncontrolled Eating (5.6 ± 2.7) and Emotional Eating (3.8 ± 1.6). The average self-esteem score according to Morris Rosenberg Self-Esteem Scale was 20.5 ± 4.7. The data acquired on the basis of the NEO-FFI Personality Inventory study demonstrated that the highest mean trait intensity was demonstrated in the subscale of conscientiousness (33.3 ± 7.2) followed by agreeableness (30.3 ± 6.2) and extraversion (29.1 ± 6.9), while the lowest intensity was demonstrated for neuroticism (22.3 ± 9.3) (Table 1).

**Table 1:** Descriptive characteristics of psychological variables in the study group of women.

Psychological variables		*M*	Me	Min–Max	Q_1_–Q_3_	SD
1.	**Personality according to NEO-FFI**					
	Neuroticism	22.30	21.00	1.0–48.00	15.00–28.00	9.30
	Extraversion	29.10	29.00	8.0–48.00	24.00–34.00	6.90
	Openness to experience	27.20	26.00	6.0–47.00	23.00–31.00	6.10
	Agreeableness	30.30	30.00	8.0–48.00	27.00–34.00	6.20
	Conscientiousness	33.30	34.00	4.0–48.00	29.00–38.00	7.20
2.	Self-esteem according to SES	20.50	20.00	4.0–30.00	18.00–23.50	4.70
3.	ORTO-15	38.00	37.50	15.0–55.00	35.00–41.00	5.40
4.	**TFEQ-13**					
	Cognitive Restraint of Eating	6.20	6.00	0.00–15.00	4.00–8.00	2.90
	Uncontrolled Eating	5.60	5.50	0.00–15.00	4.00–7.00	2.70
	Emotional Eating	3.80	4.00	0.00–9.00	3.00–5.00	1.60

M, mean; Me, median; Q_1_, lower quartile; Q_3_, upper quartile; SD, standard deviation; SES, Rosenberg Self Esteem Scale; ORTO-15, ORTO-15 questionnaire; TFEQ-13, The Three-Factor Eating Questionnaire.

Considering the extensiveness of the analyses conducted and for the sake of clarity of the study results presented in the article, the following is limited to the presentation of the statistically significant results.

### 3.1. Associations of the personality traits with risk of eating disorders

Three out of five personality traits exhibit a statistically significant effect on ORI. This effect is negative, which means that the ORI value (*p* < 0.001) decreases with an increase in the personality traits under study (N stens, E stens, and O stens). Only the personality trait of exhibits a statistically significant effect on the “Cognitive Restraint of Eating,” “Uncontrolled Eating,” and “Emotional Eating” scores (*p* < 0.001). This effect is positive, which means that score on the TFEQ-13 subscales (Table 2) increases with an increase in the personality trait under study (N stens).

**Table 2:** The relationship between the personality type of the women involved in the study and the Orthorexia Risk Index, Cognitive Restraint of Eating, Uncontrolled Eating, and Emotional Eating.

	*B*	SE	ß	−95% CI	+95% CI
**Orthorexia risk index**					
**Intercept**					
N stens	−182.60	0.042	−0.167	−0.250	−0.084
E stens	−212.88	0.048	−0.180	−0.274	−0.085
O stens	−178.61	0.043	−0.138	−0.223	−0.053
A stens	−82.72	0.046	−0.072	−0.161	0.018
C stens	23.70	0.049	0.020	−0.077	0.117
**Cognitive Restraint of Eating**					
**Intercept**					
N stens	0.19	0.042	0.234	0.152	0.317
E stens	−0.02	0.048	−0.029	−0.123	0.066
O stens	0.00	0.043	0.004	−0.081	0.089
A stens	−0.06	0.046	−0.068	−0.157	0.022
C stens	−0.07	0.050	−0.084	−0.181	0.014
**Uncontrolled Eating**					
**Intercept**					
N stens	0.11	0.043			
E stens	−0.06	0.049	0.131	0.047	0.215
O stens	0.00	0.044	−0.065	−0.162	0.031
A stens	−0.06	0.046	0.002	−0.084	0.089
C stens	−0.09	0.050	−0.067	−0.158	0.024
**Emotional Eating**					
**Intercept**					
N stens	0.07	0.044	0.129	0.043	0.214
E stens	−0.06	0.050	−0.092	−0.190	0.005
O stens	0.05	0.045	0.081	−0.006	0.169
A stens	0.04	0.047	0.071	−0.022	0.163
C stens	−0.02	0.051	−0.030	−0.131	0.070

B, unstandardized regression coefficient; SE, standard error; ß, standardized regression coefficient; CI, confidence interval; p, significance level; N, Neuroticism; E, Extraversio; O, Openness to experience; A, Agreeableness; C, Conscientiousness.

### 3.2. Associations of the level of self-esteem with risk of eating disorders

Analysis of the authors’ own research demonstrated no effect of self-esteem on ORI or on scores on the “Cognitive Restraint of Eating,” “Uncontrolled Eating,” and “Emotional Eating” scales.

### 3.3. The mediating role of self-esteem

**Figure 1:**
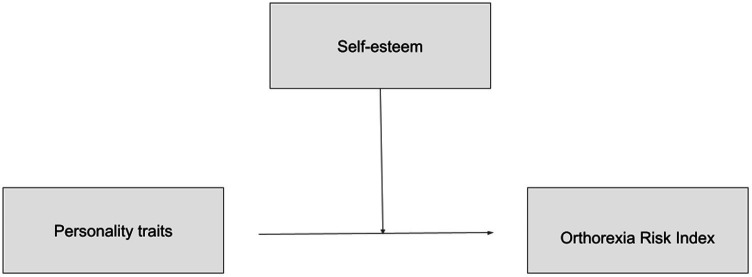
Moderation model Personality traits (NEO-FFI), Self-esteem (SES), Orthorexia Risk Index (ORTO-15).

**Figure 2:**
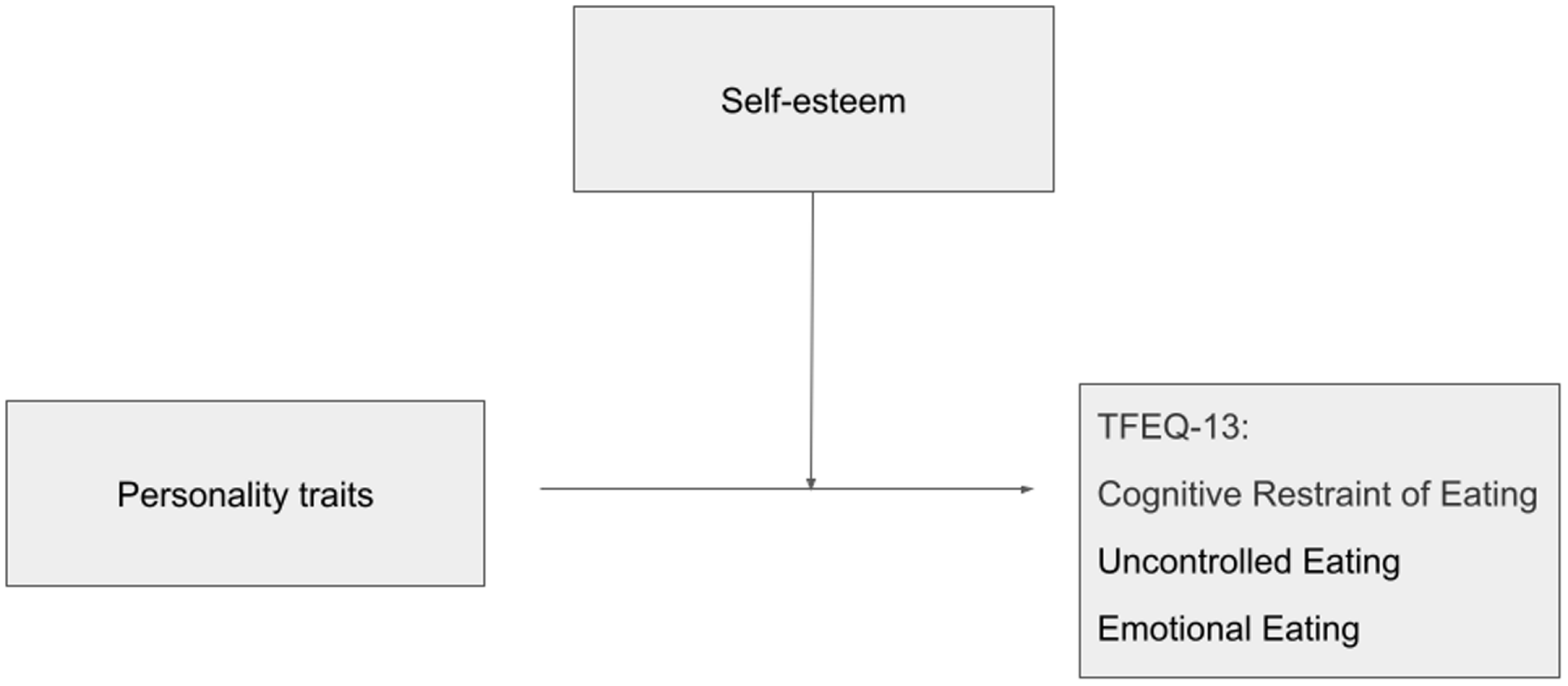
Moderation model Personality traits (NEO-FFI), Self-esteem (SES), Cognitive Restraint of Eating, Uncontrolled Eating, Emotional Eating (TFEQ-13).

**Figure 3:**
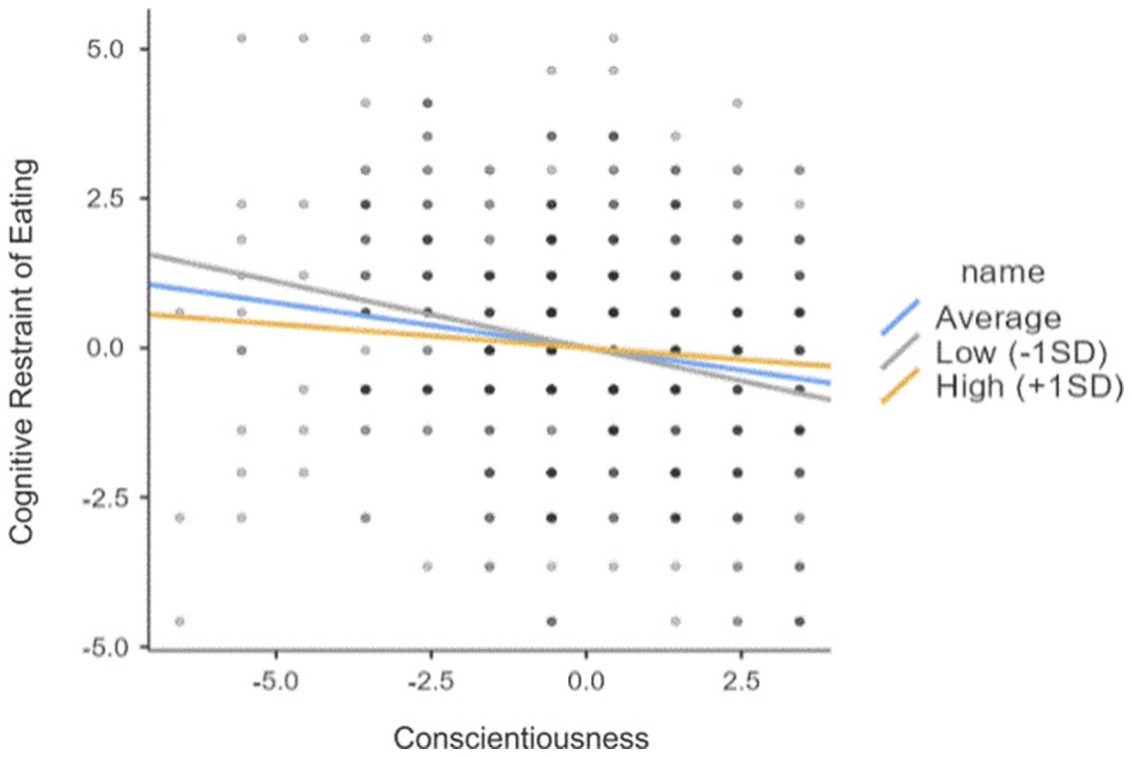
The relationship between Conscientiousness and the “Cognitive Restraint of Eating”.

In the next step, in order to check whether the relationships between women’s personality traits and the occurrence of risk of eating disorders are modified by the women’s self-esteem, a series of linear regression analyses were conducted (Figures 1, 2). Considering the extensiveness of the analyses conducted and for the sake of clarity of the study results presented in the article, the following is limited to the presentation of the statistically significant results. No moderation effect was demonstrated between self-esteem and personality traits in the effect on ORI and “Emotional Eating.” There is no moderation effect between self-esteem and personality traits (N, E, A, O) in the effect on the “Cognitive Restraint of Eating.” However, the moderation effect was demonstrated between self-esteem and the personality trait of conscientiousness in the effect on the “Cognitive Restraint of Eating” subscale score. Self-esteem was analyzed as a moderator in the relationship between conscientiousness and the “Cognitive Restraint of Eating” (Table 3, Figure 3).

**Table 3:** Analysis and the moderation effect between self-esteem and the personality trait of conscientiousness in the effect on the “Cognitive Restraint of Eating” subscale score.

		95% confidence interval			
	*b*	Lower	Upper	*Z*	*p*
C stens	−0.150	−0.220	−0.080	−4.214	<0.001
Rsbrg	−0.001	−0.022	0.020	−0.097	0.923
C stens * Rsbrg	0.010	0.000	0.019	2.040	0.041
Low (−1 SD)	−0.221	−0.320	−0.122	−4.380	<0.001
Average	−0.150	−0.220	−0.080	−4.200	<0.001
High (+ 1SD)	−0.079	−0.176	0.018	−1.590	0.111

b, unstandardized regression coefficient; CI, confidence interval.

*Moderation effect. Shows the effect of the predictor (conscientiousness) on the dependent variable (Cognitive Restraint of Eating) at different levels of the moderator (Rsbrg).

**Figure 4:**
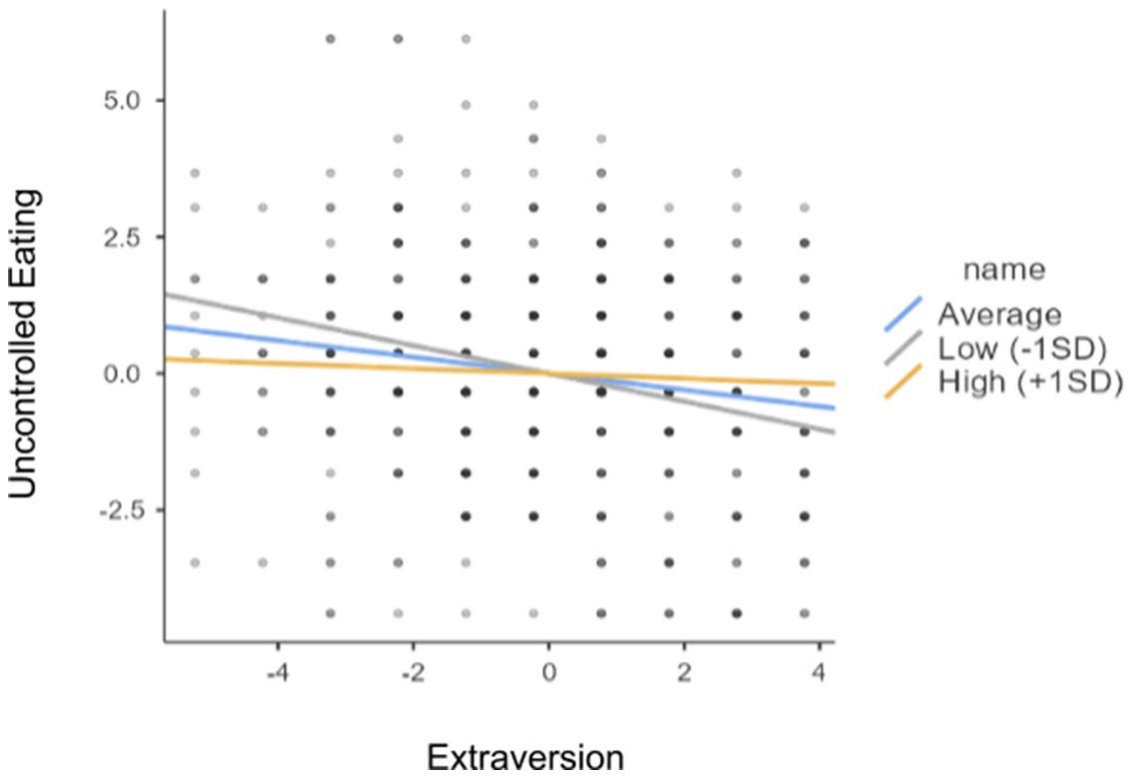
The relationship between Extraversion and the “Uncontrolled Eating”.

Analysis of the authors’ own study demonstrated no moderation effect between self-esteem and personality traits (N, O, A) in the effect on “Uncontrolled Eating.” There is a moderation effect between self-esteem and the personality trait of extraversion in the effect on the “Uncontrolled Eating” subscale score. Self-esteem was analyzed as a moderator in the relationship between extraversion and “Uncontrolled Eating” (Table 4, Figure 4).

**Table 4:** Analysis and the moderation effect between self-esteem and the personality trait of extraversion in the effect on the “Uncontrolled Eating” subscale score.

		95% confidence interval			
	*b*	Lower	Upper	*Z*	*p*
E stens	−0.151	−0.225	−0.077	−4.005	<0.001
Rsbrg	−0.005	−0.027	0.016	−0.492	0.623
E stens * Rsbrg	0.014	0.004	0.025	2.638	0.008
Low (−1 SD)	−0.256	−0.367	−0.144	−4.479	<0.001
Average	−0.151	−0.225	−0.077	−3.978	<0.001
High (+1 SD)	−0.046	−0.150	0.058	−0.872	0.383

b, unstandardized regression coefficient; CI, confidence interval.

*Moderation effect. Shows the effect of the predictor (extraversion) on the dependent variable (No control_trans) at different levels of the moderator (Rsbrg).

**Figure 5:**
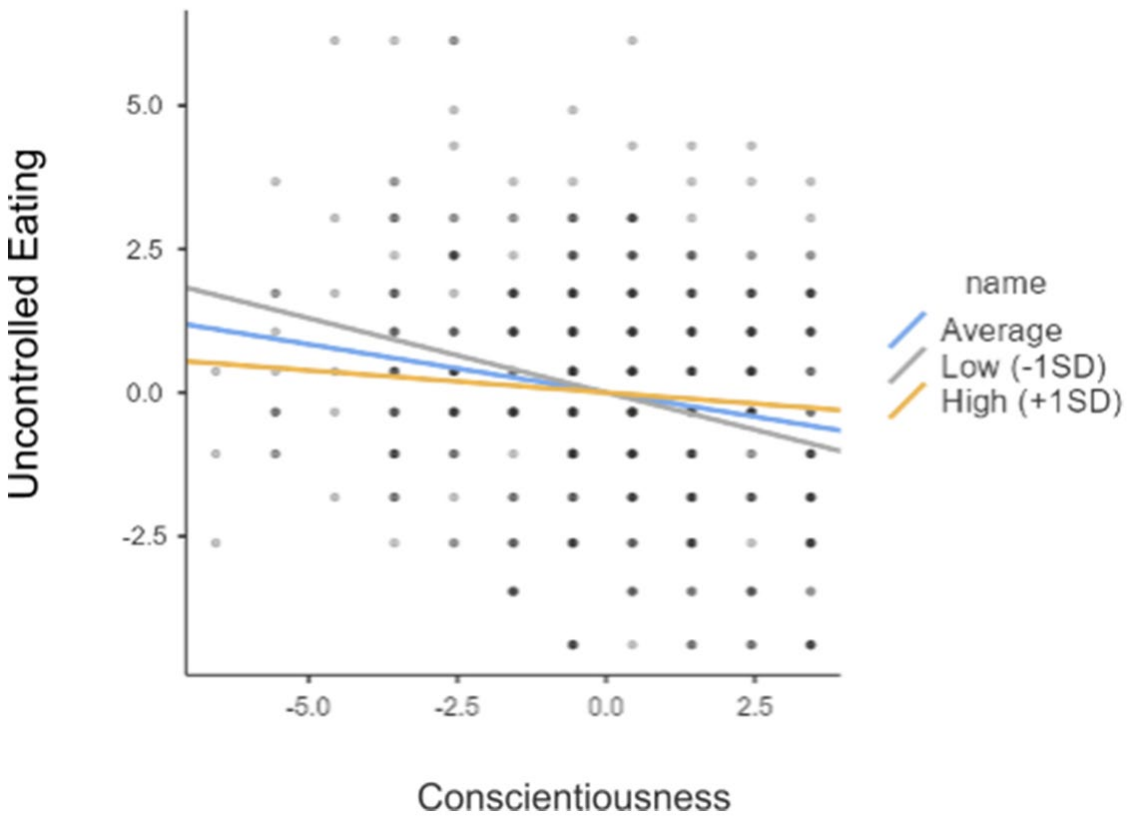
The relationship between Conscientiousness and the “Uncontrolled Eating”.

There is a moderation effect between self-esteem and the personality trait of conscientiousness in the effect on the “Uncontrolled Eating” scale score. Self-esteem was analyzed as a moderator in the relationship between conscientiousness and “Uncontrolled Eating” (Table 5, Figure 5).

**Table 5:** Analysis and the effect of moderation between self-esteem and the personality trait of conscientiousness in the effect on the Uncontrolled Eating subscale score.

		95% confidence interval			
	*b*	Lower	Upper	*Z*	*p*
C stens	−0.168	−0.240	−0.096	−4.547	<0.001
Rsbrg	−0.011	−0.032	0.011	−0.990	0.322
C stens * Rsbrg	0.012	0.003	0.022	2.506	0.012
Low (−1 SD)	−0.259	−0.362	−0.156	−4.930	<0.001
Average	−0.168	−0.241	−0.095	−4.520	<0.001
High (+1 SD)	−0.077	−0.178	0.024	−1.500	0.133

b, unstandardized regression coefficient; CI, confidence interval.

*Moderation effect. Shows the effect of the predictor (conscientiousness) on the dependent variable (No control_trans) at different levels of the moderator (Rsbrg).

## 4. Discussion

Eating disorders (ED) are psychological problems, although their effects also extend to the physical and social spheres. ED represent an increasingly common problem, especially in the Western culture, which promotes a slim body image, while the pace of life, demands, and peer pressure are increasing (46). Men tend to suppress their emotions more than women. Women tend to eat emotionally more often than men; they are also more likely to control the eating process. Individuals with a low BMI exhibit a greater degree of emotional suppression than individuals of normal or high body weight. Therefore, mental and behavioral problems in the female population should be studied more extensively. In order to deepen our understanding of these findings, we should start by highlighting that sex appears to be an important determinant of non-normative eating behaviors (28).

To the best of our knowledge, this is the first study to investigate the moderating role of self-esteem in the relationships between personality traits and the occurrence of risk of eating disorders. The results obtained in this study provided the following contribution to the literature.

Low self-esteem, defined as attitudes toward and evaluation of one’s own self-worth, is a central risk for disturbances in eating and body image. The authors’ own study demonstrated no effect of self-esteem on the occurrence of risk of eating disorders while indicating that self-esteem modifies the relationships between certain Big Five model personality traits and the occurrence of risk of eating disorders. Analysis of the authors’ own research showed the existence of moderation between self-esteem and conscientiousness in the effect on Uncontrolled Eating and Cognitive Restraint of Eating, and extraversion in the effect on Uncontrolled Eating. The study demonstrated a lack of moderating role of self-esteem between personality traits and ORI and Emotional Eating, between neuroticism, extraversion, agreeableness, and openness to experience in the effect on Cognitive Restraint of Eating, and between neurotics, openness to experience, and agreeableness in the effect on Uncontrolled Eating. Low self-esteem, pessimism about life, a sense of helplessness, and an excessive need for achievements, and striving for perfectionism trigger negative emotions and the need for comfort, often satisfied by eating food. In neurotic individuals who are prone to experiencing negative emotions and in introverted individuals who isolate themselves from others, eating may serve the function of alleviating unpleasant emotional states and the feeling of loneliness (47). Individuals with eating disorders may also encounter difficulties in managing their eating behavior if the basic psychological factors, such as lower self-esteem, are ignored or repressed. In order to effectively address the issue of changes in eating behaviors, mental functioning variables appear to be important proximal clinical targets as compared to the personality traits that are more difficult to modify. Interventions that focus directly on body esteem and mood regulation can be part of an interdisciplinary nutrition management model (48).

The analysis results showed that certain personality dimensions are reflected in eating behaviors. A higher level of neuroticism was identified as an important predictor of higher results for orthorexia, Cognitive Restraint of Eating, Uncontrolled Eating, and Emotional Eating. It was also demonstrated that the orthorexia risk index decreased with increased extraversion and openness to experience. Conscientiousness was also positively linked to the moderation effect between self-esteem in the effect on uncontrolled eating. What is more, the results of this study stress the effect of psychological factors in the observed links between personality traits and certain, but not all, eating behaviors measured. The earlier studies confirm the results of our study. Many authors identified the personality aspects related to neuroticism as important predictors of Uncontrolled Eating and Cognitive Restraint of Eating (49, 50). The study by Forester identified a significant negative correlation between the ORTO-15 results and Neuroticism, as well as a negative correlation between the ORTO-15 results and openness to experience. However, no correlation was found between the ORTO-15 results and conscientiousness, extraversion, and agreeableness (51).

As for neuroticism, the hypothesis can be formulated that a high degree of susceptibility to stress and pronounced sensitivity to negative effects that characterize this trait can, at least partially, act as a “trigger” for eating disorders. Therefore, such behavior will become a “releaser of emotional distress” and a way of coping with stress. Following this line of argument, even timidly, the possible route of influence could be negative emotionality—stress—eating disorders. As demonstrated in the literature, women characterized by a high level of neuroticism may exhibit difficulties related to their general psychosocial functioning (52) and present eating disorders (53). It can therefore be suggested that neuroticism may predispose to certain mental health difficulties, such as dissatisfaction with one’s appearance. These psychological traits may eventually be associated with non-normative eating behaviors. According to the previous studies (28, 50), it appears important that in order to maintain conscious control over the intake of food, the presence of conscientiousness may be necessary, which is in line with the definition of Cognitive Restraint of Eating, which implies self-control. To support this explanation, it is worth stressing that neuroticism was consistently observed in women with eating disorders (54).

Agreeableness had previously been identified as a less important personality trait when compared to conscientiousness and neuroticism in understanding eating behaviors (51), as confirmed by the authors’ own study. In contrast, a meta-analysis showed a negative relationship between different psychological symptoms and agreeableness (52), which may suggest that a higher level of agreeableness can be beneficial for mental health and therefore be associated with healthier behavioral factors. However, the women in the study sample were characterized by a moderately high agreeableness result as compared to the standard reference group of women (17). This peculiarity limits the generalization of the results observed, but it may emphasize that a higher level of agreeableness appears to be an important trait in women with selected eating disorders (28).

In addition to physiological or social functions, eating food also has a psychological dimension, particularly in terms of regulating emotional states (eating food in moments of sadness, joy, or boredom). An important role is also served here by the cultural messages, especially media coverage—the need to control the food consumed and, consequently, control over one’s body and its weight and dimensions. Higher results for Emotional Eating were observed in individuals with lower self-esteem, who perceived themselves as not very physically fit, and when the study subjects were on a diet. In the Uncontrolled Eating subscale, higher results were noted in individuals who perceived themselves to be in poor physical condition (55). As the above study show, not only is Emotional Eating related to the quality and quantity of the food consumed but also to the lack of ability to cope with stress, the image of one’s body, and overall self-esteem (56).

The earlier studies confirm the relationships between personality traits and eating behavior. A higher neuroticism level and a lower conscientiousness level (51), as well as the presence of emotional instability (57), were associated with Emotional Eating. Similar results were observed in eating behaviors where neuroticism explains 17% of Uncontrolled Eating (49). Meanwhile, mental functioning traits such as self-esteem and the perception of body image were previously related to body weight status (56) and may be significant correlates of eating behaviors (57). Moreover, relationships were noted between personality traits, particularly neuroticism, and body image disturbances (58), which is in line with the definition of neuroticism, i.e., a tendency to experience negative emotions. Following these observations, it appears important to consider specific psychological variables in the study into relationships between personality traits, self-esteem, and eating behaviors.

The present findings highlight the most important factors to be taken into account when assessing the indices of orthorexia, Uncontrolled Eating and Emotional Eating in future research. In addition to the several factors considered in this study based on the literature, future research may take into account other factors related to eating behavior dimensions, i.e., satisfaction with body weight, nutritional knowledge, and one’s body image. In order to better understand the links between the eating behavior dimensions and their causal factors, longitudinal studies are needed (59).

The results of this study may pave the way for clients to deal with eating disorders. Since determining personality type in many cases explains the causes of mental illness and how it spreads, it may also be helpful to take into account this issue regarding unhealthy eating behaviors (60).

## 5. Limitations

The analyses presented in this study identified certain limitations and implications for professional practice. This study represents one of the first such large-scale studies conducted among women in Poland. The main strengths of the study were the large sample size and the consideration of important and reliable psychometric tools. Moreover, the use of standardized tools enriched the data presented. In view of the direct impact of neuroticism on the pattern of behavior, it seems appropriate to design interventions aimed at reducing the impact of this personality trait on psychosocial functioning and non-normative eating behaviors. This study also shows certain potential limitations, which, hopefully, will be overcome by future research, including the lack of data on the occurrence of risk of disorders in psychosocial functioning, which could be of importance for differentiating the effect of variables on the occurrence of risk of non-normative eating behaviors. The use of larger samples from different socio-cultural contexts and paying attention to other interesting variables (e.g., income level or social class) are only some of the aspects to be considered in the future. Another limitation may be problems with the generalizability of the data to other cultures. The limitation is also a cross-sectional, correlational nature of our project, with moderation analyses based on the data collected, and limits the ability to identify causal relationships. Limitations can also refer to the use of standardized clinical interviews in accordance with official manuals for future studies. The study was based solely on measurements of self-descriptive constructs. Autobiographically derived information can be essential to gain an overall picture of a particular person’s behavior. Despite its limitations, this study provides important findings and can be a starting point for broader research aimed at determining the moderating role of self-esteem in the relationships between personality traits among Polish women and the occurrence of risk of eating disorders.

## 6. Recommendations for further research

An assessment of eating behaviors and the related factors, based on self-report tools, can be distorted due to the desire to be perceived by society as attractive, i.e., a tendency to avoid criticism and provide more socially acceptable answers. This may result in overestimating healthy behaviors and underestimating undesirable ones. Knowledge of the effect of social desirability on self-reported eating behaviors and their links with other factors may be useful in improving the accuracy of dietary assessment and developing effective strategies for preventing eating and body weight disorders. This study provides important findings and can be a starting point for broader research aimed at determining the moderating role of self-esteem in the relationships between personality traits among Polish women and the occurrence of risk of eating disorders.

## 7. Conclusion

Self-esteem was not a predictor of the occurrence of risk of eating disorders and plays a moderating role in the relationship between certain Big Five model personality traits and the occurrence of risk of eating disorders. A higher level of neuroticism was identified as an important predictor of higher results for orthorexia, Cognitive Restraint of Eating, Uncontrolled Eating, and Emotional Eating. It was also demonstrated that the orthorexia risk index decreased with increased extraversion and openness to experience. The results of this study suggest that neuroticism, conscientiousness, agreeableness, and mental functioning are important factors that contribute to a better understanding of eating behaviors. Measurement of personality traits can be useful to identify women who can be prone to non-normative eating behaviors, which, in turn, may hinder their attempts to control their body weight. In addition, the results of this study suggest that eating behaviors and psychological factors should be included in psychological interventions in the treatment of eating disorders. The clinical goal can be considered to be an improvement in non-normative eating behaviors, such as a reduction in overeating episodes or eating less frequently in the absence of a hunger feeling. In order to assist these individuals in their attempts to achieve healthy behaviors, variables related to mental functioning can be then identified as important goals to support individuals in their efforts to change health behaviors by achieving better mental well-being.

## Data availability statement

The raw data supporting the conclusions of this article will be made available by the authors, without undue reservation.

## Ethics statement

The studies involving humans were approved by Bioethics Committee of the Pomeranian Medical University in Szczecin (Resolution No KB-0012/518/12/16). The studies were conducted in accordance with the local legislation and institutional requirements. The participants provided their written informed consent to participate in this study.

## Author contributions

KR: Conceptualization, Methodology, Visualization, Writing – original draft, Writing – review & editing. AC: Conceptualization, Data curation, Formal analysis, Project administration, Visualization, Writing – original draft, Writing – review & editing. DS-M: Resources, Writing – original draft. MP: Validation, Visualization, Writing – original draft. EK: Supervision, Writing – original draft. MK: Investigation, Writing – review & editing. EG: Funding acquisition, Software, Writing – original draft.

## References

[1] Kowalkowska J Poínhos R. Eating behavior among university students: associations with age, socioeconomic status, physical activity, body mass index, waist to height ratio, and social desirability. Nutrients. (2021) 13:3622. doi: 10.3390/nu13103622, PMID: 34684623 PMC8541155

[2] Czarnocinska J Wadolowska L Lonnie M Kowalkowska J Jezewska-Zychowicz M Babicz-Zielinska E. Regional and socioeconomic variations in dietary patterns in a representative sample of young polish females: a cross-sectional study (GEBaHealth project). Nutr J. (2020) 19:26. doi: 10.1186/s12937-020-00546-8, PMID: 32245487 PMC7126359

[3] Koletzko B Brands B Poston L Godfrey K Demmelmair H. Early nutrition programming of long-term health. Proc Nutr Soc. (2012) 71:371–8. doi: 10.1017/S0029665112000596 22703585

[4] Ogden J. Nutrition psychology. From healthy to disordered eating behaviors. Kraków: UJ (2011).

[5] Jankowska J Helbin J. Various means of rewarding oneself in positive and negative emotional states. Probl Hig Epidemiol. (2010) 91:425–7.

[6] Ziółkowska B. Psychosocial aspects of non-normative body weight. Poznań: UAM (2014).

[7] Leszczyńska S Błażejewska K Lewandowska-Klafczyńska K Rycielski P. Emotions and eating behavior in women aged 18-30. Endokr Otyłość Zab Przem Mat. (2011) 7:167–71.

[8] Wieczorkowska-Wierzbińska G. Psychological limitations. Warszawa: UW (2011).

[9] Łucka I Janikowska-Hołoweńko D Domarecki P Plenikowska-Ślusarz T Domarecka M. Orthorexia nervosa-a separate clinical entity, a part of eating disorder spectrum or another manifestation of obsessive-compulsive disorder. Psychiatr Pol. (2019) 53:371–82. doi: 10.12740/PP/OnlineFirst/85729 31317964

[10] Koven NS Abry AW. The clinical basis of orthorexia nervosa: emerging perspectives. Neuropsychiatr Dis Treat. (2015) 11:385–94. doi: 10.2147/NDT.S61665, PMID: 25733839 PMC4340368

[11] Mathieu J. What is orthorexia? J Am Diet Assoc. (2005) 105:1510–2. doi: 10.1016/j.jada.2005.08.021 16183346

[12] Gałecki P Święcicki Ł. Diagnostic criteria with DSM-5®. Desk reference. Wrocław: Edra Urban & Partner (2015).

[13] Pużyński S Wciórka J. Classification of mental and behavioral disorders in ICD-10. Research diagnostic criteria. 5th chapter. Kraków: Vesalius University Medical Publishing House (1998).

[14] Gaebel W Zielasek J Reed GM. Mental and behavioral disorders in ICD-11: concepts, methodologies and current status. Psychiatr Pol. (2017) 51:169–95. doi: 10.12740/pp/69660 28581530

[15] Wadden TA Womble LG Stunkard AJ Anderson DA. Psychosocial consequences of obesity and weight loss. (2002)

[16] Mora F Fernandez Rojo S Banzo C Quintero J. The impact of self-esteem on eating disorders. European Psychiatr. (2017) 41:S558–8. doi: 10.1016/j.eurpsy.2017.01.802

[17] Costa PT Jr McCrae RR. Revised NEO personality Inventory (NEO-PI-R) and NEO five-factor Inventory (NEO-FFI) professional manual. Odessa, FL: Psychological Assessment Resources (1992).

[18] Varga M Dukay-Szabó S Túry F. Evidence and gaps in the lit-erature on orthorexia nervosa. Eat Weight Disord. (2013) 18:103–11. doi: 10.1007/s40519-013-0026-y, PMID: 23760837

[19] Donini LM Marsili D Graziani MP. Orthorexia nervosa: a pre-liminary study with a proposal for diagnosis and an attempt to measure the dimension of the phenomenon. Eat Weight Disord. (2004) 9:151–7. doi: 10.1007/BF03325060 15330084

[20] Michalska A Szejko N Jakubczyk A. Non-specific eating disorders – a subjective review. Psychiatr Pol. (2016) 50:497–507. doi: 10.12740/pp/59217, PMID: 27556109

[21] Saddichha S Babu GN Chandra P. Orthorexia nervosa presenting as prodrome of schizophrenia. Schizophr Res. (2012) 134:110. doi: 10.1016/j.schres.2011.10.017, PMID: 22088557

[22] Oberle CD Samaghabadi RO Hughes EM. Orthorexia nervosa: assessment and correlates with gender, BMI, and personality. Appetite. (2017) 108:303–10. doi: 10.1016/j.appet.2016.10.021, PMID: 27756637

[23] Arusoğlu G Kabakçi E Köksal G. Orthorexia nervosa and adaptation of ORTO-11 into Turkish. Turk Psikiyatri Derg. (2008) 19:283–91. PMID: 18791881

[24] Van de Mortel TF. Faking it: social desirability response bias in self-report research. Aust J Adv Nurs. (2008) 25:40–8.

[25] Adams SA Matthews CE Ebbeling CB Moore CG Cunningham JE Fulton J. The effect of social desirability and social approval on self-reports of physical activity. Am J Epidemiol. (2005) 161:389–98. doi: 10.1093/aje/kwi054 15692083 PMC2958515

[26] Mossavar-Rahmani Y Tinker LF Huang Y Neuhouser ML McCann SE Seguin RA. Factors relating to eating style, social desirability, body image and eating meals at home increase the precision of calibration equations correcting self-report measures of diet using recovery biomarkers: findings from the Women’s health initiative. Nutr J. (2013) 12:63. doi: 10.1186/1475-2891-12-63 23679960 PMC3658913

[27] Poínhos R Oliveira BMPM Correia F. Eating behavior in Portuguese higher education students: the effect of social desirability. Nutrition. (2015) 31:310–4. doi: 10.1016/j.nut.2014.07.008, PMID: 25592009

[28] Provencher V Drapeau V Tremblay A Després JP Lemieux S. Eating behaviors and indexes of body composition in men and women from the Québec family study. Obes Res. (2003) 11:783–92. doi: 10.1038/oby.2003.109, PMID: 12805400

[29] McLean JA Barr SI. Cognitive dietary restraint is associated with eating behaviors, lifestyle practices, personality characteristics and menstrual irregularity in college women. Appetite. (2003) 40:185–92. doi: 10.1016/S0195-6663(02)00125-3, PMID: 12781168

[30] Stunkard AJ Messick S. The three-factor eating questionnaire to measure dietary restraint, disinhibition and hunger. J Psychosom Res. (1985) 29:71–83. doi: 10.1016/0022-3999(85)90010-8 3981480

[31] McLean JA Barr SI Prior JC. Dietary restraint, exercise, and bone density in young women: are they related? Med Sci Sports Exerc. (2001) 33:1292–6. doi: 10.1097/00005768-200108000-00008, PMID: 11474329

[32] McLean JA Barr SI Prior JC. Cognitive dietary restraint is associated with higher urinary cortisol excretion in healthy premenopausal women. Americ J Clin Nutr. (2001) 73:7–12. doi: 10.1093/ajcn/73.1.7, PMID: 11124742

[33] de Lauzon B Romon M Deschamps V Lafay L Borys JM Karlsson J. The three-factor eating questionnaire-R18 is able to distinguish among different eating patterns in a general population. J Nutr. (2004) 134:2372–80. doi: 10.1093/jn/134.9.2372 15333731

[34] Valladares M Durán E Matheus A Durán-Agüero S Obregón AM Ramírez-Tagle R. Association between eating behavior and academic performance in university students. J Am Coll Nutr. (2016) 35:699–703. doi: 10.1080/07315724.2016.1157526 27736367

[35] Löffler A Luck T Then FS Luck-Sikorski C Pabst A Kovacs P. Effects of psychological eating behaviour domains on the association between socio-economic status and BMI. Public Health Nutr. (2017) 20:2706–12. doi: 10.1017/S1368980017001653, PMID: 28735590 PMC10261281

[36] de Castro JM. The relationship of cognitive restraint to the spontaneous food and fluid intake of free-living humans. Physiol Behav. (1995) 57:287–95. doi: 10.1016/0031-9384(94)00229-X, PMID: 7716205

[37] Tasca GA Demidenko N Krysanski V Bissada H Illing V Glick M. Personality dimensions among women with an eating disorder: towards reconceptualizing DSM. Eur Eat Disord Rev. (2009) 17:281–9. doi: 10.1002/erv.938, PMID: 19421961

[38] Bollen E Wojciechowski FL. Anorexia nervosa subtypes and the big five personality factors. Eur Eat Disord Rev. (2004) 12:117–21. doi: 10.1002/erv.551

[39] Ghaderi A Scott B. The big five and eating disorders: a prospectivestudy in the general population. Eur J Personal. (2000) 14:311–23. doi: 10.1002/1099-0984(200007/08)14:4

[40] Elfhag K Morey LC. Personality traits and eating behavior in the obese: poor self-control in emotional and external eating but personality assets in restrained eating. Eat Behav. (2008) 9:285–93. doi: 10.1016/j.eatbeh.2007.10.003, PMID: 18549987

[41] Zawadzki B Strelau J Szczepaniak P Costa Śliwińska M. McCrae's NEO-FFI Inventory Personality. Polish adaptation. Coursebook. Laboratory of Psychological Tests of the Polish Psychological Association, Warszawa (1998) 13–133.

[42] Łaguna M Lachowicz-Tabaczek K Dzwonkowska I. Morris' SES self-esteem scale Rosenberg - polish adaptation of the method. Psychologia Społeczna. (2007) 2:164–76.

[43] Rosenberg M. Society and adolescent self-image. Revised ed. Middletown, CT: Wesleyan University Press (1989).

[44] Stochel M Janas-Kozik M Zejda JE Hyrnik J Jelonek I Siwiec A. Questionnaire validation ORTO-15 in a group of urban youth aged 15–21. Psychiatr Pol. (2015) 49:119–34. doi: 10.12740/PP/25962 25844415

[45] Dzielska A Mazur J Małkowska-Szkutnik A Kołoło H. Adaptation of the polish version of the three-factor eating questionnaire (TFEQ-13) among school youth in population studies. Problemy Higieny i Epidemiologii. (2009) 90:362–9.

[46] Jabłońska E Błądkowska K Bronkowska M. Eating disorders as a health and psychosocial problem. Kosmos. (2019) 68:121–32. doi: 10.36921/kos.2019_2489

[47] Pietrzykowska E Wierusz-Wysocka B. Psychological aspects of overweight, obesity and slimming. Pol Merk Lek. (2008) 24:472. 18634399

[48] Bégin C Gagnon-Girouard MP Provencher V Lemieux S. Traitement de l'obésité: Soutenir l'individu dans l'appropriation de sa démarche. Can Psychol. (2006) 47:316–32. doi: 10.1037/cp2006021

[49] Kaye W Gendall K Strober M. Serotonin neuronal function and selective serotonin reuptake inhibitor treatment in anorexia and bulimia nervosa. Biol Psychiatry. (1998) 44:825–38. doi: 10.1016/S0006-3223(98)00195-4, PMID: 9807638

[50] Heaven PC Mulligan K Merrilees R Woods T Fairooz Y. Neuroticism and conscientiousness as predictors of emotional, external, and restrained eating behaviors. Int J Eat Disord. (2001) 30:161–6. doi: 10.1002/eat.1068, PMID: 11449449

[51] Forester DS. Examining the relationship between orthorexia nervosa and personality traits. (Doctoral dissertation) (2014).

[52] Malouff JM Thorsteinsson EB Schutte NS. The relationship between the five-factor model of personality and symptoms of clinical disorders: a meta-analysis. J Psychopathol Behav Assess. (2005) 27:101–14. doi: 10.1007/s10862-005-5384-y

[53] Cassin SE von Ranson KM. Personality and eating disorders: a decade in review. Clin Psychol Rev. (2005) 25:895–916. doi: 10.1016/j.cpr.2005.04.012 16099563

[54] Bulik CM Sullivan PF Kendler KS. Medical and psychiatric morbidity in obese women with and without binge eating. Int J Eat Disord. (2002) 32:72–8. doi: 10.1002/eat.10072 12183948

[55] Jáuregui-Lobera I García-Cruz P Carbonero-Carreño R Magallares A Ruiz-Prieto I. Psychometric properties of Spanish version of the three-factor eating questionnaire-R18 (Tfeq-Sp) and its relationship with some eating-and body image-related variables. Nutrients. (2014) 6:5619–35. doi: 10.3390/nu6125619, PMID: 25486370 PMC4276988

[56] Blomquist KK Grilo CM. Predictive significance of changes in dietary restraint in obese patients with binge eating disorder during treatment. Int J Eat Disord. (2011) 44:515–23. doi: 10.1002/eat, PMID: 20957705 PMC3025064

[57] Appleton KM McGowan L. The relationship between restrained eating and poor psychological health is moderated by pleasure normally associated with eating. Eat Behav. (2006) 7:342–7. doi: 10.1016/j.eatbeh.2005.11.008, PMID: 17056410

[58] Tylka TL Subich LM. Examining a multidimensional model of eating disorder symptomatology among college women. J Couns Psychol. (2004) 51:314–28. doi: 10.1037/0022-0167.51.3.314

[59] Bond MJ McDowell AJ Wilkinson JY. The measurement of dietary restraint, disinhibition and hunger: an examination of the factor structure of the three factor eating questionnaire (TFEQ). Int J Obes Relat Metab Disord. (2001) 25:900–6. doi: 10.1038/sj.ijo.0801611, PMID: 11439306

[60] Reshadat S Zakiei A Hatamin P Bagheri A Rostami S Komasi S. A study of the correlation of personality traits (neuroticism and psychoticism) and self-efficacy in weight control with unhealthy eating behaviors and attitudes. Ann Med Health Sci Res. (2017) 7:32–8.

