# Supplementary material for: Personality traits and risk of eating disorders among Polish women: the moderating role of self-esteem
Source: Front Psychiatry. 2023 Oct 31;14:1281898. doi: 10.3389/fpsyt.2023.1281898 (PMC10644397; doi:10.3389/fpsyt.2023.1281898)
Supplement: Supplementary file 1 [file Table_1.docx]

**Table S1**. General sociodemographic characteristics of the study group (N = 556).

| Sociodemographic variables | | n | % |
| --- | --- | --- | --- |
| Education | Lower (secondary, vocational, elementary) | 287 | 51.6 |
|  | Higher | 269 | 48.4 |
| Marital status | Single (maiden, divorced, widow) | 186 | 34.5 |
|  | in a formal / informal relationship | 370 | 66.5 |
| Place of residence | < 100.000 inhabitants | 293 | 52.7 |
|  | ≥ 100.000 inhabitants | 263 | 47.3 |
| Professional activity | Professionally active | 496 | 89.2 |
|  | Professionally inactive | 60 | 10.8 |
| n - number of cases, % - percentage of the total study group | | | |
